# Phenotypic profiling of Pathogen Box compounds MMV667494 and MMV028694 in bloodstream-form *Trypanosoma brucei brucei*

**DOI:** 10.3389/ebm.2026.10979

**Published:** 2026-06-12

**Authors:** Pearl Ihuoma Akazue, Neils Ben Quashie, Sue Vaughan, Harry P. de Koning, Theresa Manful Gwira

**Affiliations:** 1 West African Centre for Cell Biology of Infectious Pathogens, University of Ghana, Accra, Ghana; 2 Department of Biochemistry, Cell and Molecular Biology, University of Ghana, Accra, Ghana; 3 Department of Biochemistry, Faculty of Life Sciences, University of Benin, Benin City, Nigeria; 4 Centre for Tropical Clinical Pharmacology and Therapeutics, University of Ghana Medical School, Accra, Ghana; 5 Department of Biological and Medical Sciences, Oxford Brookes University, Oxford, United Kingdom; 6 School of Infection and Immunity, University of Glasgow, Glasgow, United Kingdom

**Keywords:** African trypanosomiasis, antitrypanosomal activity, mitochondrial reactive oxygen species, mitochondrial stress, Pathogen Box

## Abstract

Open-access drug discovery platforms have accelerated hit identification and lead prioritization across multiple diseases and enable systematic repurposing of bioactive compounds beyond their original indications. However, there remains a need for new chemotypes for African trypanosomiasis with improved efficacy and resilience to emerging drug resistance. In this study, we evaluated the antitrypanosomal potential and cellular effects of two Pathogen Box compounds, MMV667494 and MMV028694. The compounds were selected through a resazurin-based *in vitro* phenotypic viability screen that measures metabolic activity as a proxy for parasite viability against bloodstream-form *Trypanosoma brucei brucei*. To explore cellular phenotypes consistent with potential mechanisms of action, we applied cytological profiling using flow cytometry- and microscopy-based assays, including Annexin V/propidium iodide staining, cell-cycle DNA-content analysis, mitochondrial membrane potential (TMRE), and mitochondrial reactive oxygen species (MitoSOX) measurements. Both MMV667494 and MMV028694 (IC_50_ = 0.44 ± 0.05 µM and 0.33 ± 0.03 µM, respectively) displayed sub-micromolar antitrypanosomal potency and preferential toxicity toward trypanosomes over mammalian cells (selectivity indices >10). Growth profiling demonstrated dose-dependent inhibition of parasite proliferation, with evidence of trypanocidal activity at higher concentrations and longer exposure times. Treatment resulted in increased populations of phosphatidylserine-exposed and membrane-compromised cells, which is consistent with apoptosis-like phenotypes in trypanosomes. Although both compounds induced mitochondrial membrane depolarization in treated *T. b. brucei* cells, this effect was observed predominantly in a subpopulation of cells and is therefore unlikely to represent the primary cause of cell death. Increased mitochondrial production of reactive oxygen species and altered cell-cycle progression were also observed, which might indicate disruption of key cellular processes. These findings shows that MMV667494 and MMV028694 are selective antitrypanosomal compounds and their activities are associated with induce apoptosis-like features, cell-cycle disruption, and mitochondrial stress signatures in bloodstream-form *T. b. brucei*. These findings provide phenotypic insights into the activity of the compounds, warranting further target deconvolution and optimization, although validation in human-infective subspecies and *in vivo* systems will be required.

## Impact statement

New chemotherapeutics for African trypanosomiasis with improved efficacy and resilience to drug resistance are still needed despite recent significant advances in drug discovery for the disease. This study investigated the possible mode of action of two promising Pathogen Box compounds, MMV667494 and MMV028694, using phenotypic profiling. The compounds were identified through a resazurin-based *in vitro* cell viability screen. We show that MMV667494 and MMV028694 are selective antitrypanosomal compounds that induce apoptosis-like phenotypes, cell-cycle disruption, and mitochondrial stress signatures in bloodstream-form *Trypanosoma brucei brucei*. These data provide evidence that these compounds could be repurposed for African trypanosomiasis chemotherapy.

## Introduction

African trypanosomiasis is a two-staged parasitic disease, caused by the protozoa of the genus *Trypanosoma*, that affects humans and animals imposing severe public-health and socioeconomic consequences in endemic regions of sub-Saharan Africa [1–3]. Human African trypanosomiasis (HAT), also known as sleeping sickness, is caused by two subspecies of *Trypanosoma brucei*: *T. b. gambiense* and *T. b. rhodesiense*. The early (haemolymphatic) stage is often characterized by nonspecific symptoms such as intermittent fever, which can delay diagnosis, allowing progression to the late (meningoencephalitic) stage, a condition that is invariably fatal if untreated [4]. In parallel, animal African trypanosomiasis (AAT), caused by several *Trypanosoma species*, poses a significant constraint on livestock productivity and food security. Unlike HAT, AAT is not confined to the geographical distribution of the tsetse fly; species such as *T. vivax* and *T. evansi* have adapted to mechanical transmission by other biting flies, while *T. equiperdum* is transmitted sexually among equines [5].

Current chemotherapy for African trypanosomiasis relies on a few repertoires of drugs, many of which are decades old, stage-specific, difficult to administer, or associated with significant toxicity, and continuous use is seriously threatened by rising levels of drug resistance [6, 7]. Although recent advances have led to the introduction of oral agents such as fexinidazole and promising late-stage candidates [8–10], substantial gaps remain. These include the risk of emerging drug resistance, limited therapeutic options for late-stage *T. b. rhodesiense* HAT, inadequate treatments for AAT, and the challenge posed by parasite populations adapted to distinct host tissues and organs [7, 11–18].

Drug discovery efforts aim to identify hit compounds that can be optimized into viable therapeutic candidates, traditionally drawing heavily from natural products, which have historically inspired many successful antiparasitic drugs [19–22]. However, the relative decline in natural-product-based discovery pipelines has been attributed to factors such as complex chemical architectures with high sp^3^ character and multiple stereocenters, challenging synthesis and derivatization, batch-to-batch variability, and constraints on scalable production [19, 23].

In contrast, synthetic compound libraries enable rapid, scalable (semi)synthesis and systematic optimization of hits and leads via scaffold-hopping and expansion strategies. Public–private partnerships have further accelerated these efforts by providing open access compound libraries. For instance, the Medicines for Malaria Venture (MMV) provides open-access compound libraries, including the “Pathogen Box” used in this study. The Pathogen Box is a collection of 400 diverse, drug-like molecules (including 26 reference compounds) with documented activity against pathogens that cause neglected tropical diseases. Several Pathogen Box compounds have shown promising activity against parasites beyond their original target indications [24–30]. Importantly, only a subset of these compounds has documented activity against kinetoplastids, highlighting the potential for systematic repurposing of the remaining compounds for antitrypanosomal drug discovery.

In an earlier study, we identified promising antitrypanosomal constituents from herbal preparation that were originally used in the treatment of schistosomiasis rather than African trypanosomiasis, demonstrating the feasibility of repurposing treatments developed for other neglected tropical diseases [31, 32]. Similarly, de Brito et al. reported the antischistosomal activity of diminazene aceturate, one of the standard antitrypanosomal drugs [33], further demonstrating the potential of repurposing efforts in facilitating the discovery and development of new treatments for neglected diseases [34]. In this study, we screened selected compounds from the MMV pathogen box for activity against bloodstream form *T. b. brucei* and performed phenotypic profiling to investigate cellular responses associated with compound exposure. Using cell-based phenotypic assays coupled with flow cytometry and confocal microscopy, we examined the effects of two selected compounds, MMV667494 and MMV028694, on mitochondrial function and cell cycle progression. While this study focuses on *T. b. brucei* as a model organism, the findings provide preliminary insights that may inform future investigations in human-infective subspecies.

## Materials and methods

### General compound information and compound selection

Stock solutions of individual test compounds in 100% DMSO (10 µL of 10 mM solution per compound) were provided by the Medicines for Malaria Venture (MMV;^1^). The compounds originated from the MMV Pathogen Box, an open-access compound collection of 400 structurally diverse, drug-like molecules with activity against pathogens responsible for neglected tropical diseases. The pathogen box is distributed across five 96-well plates, each containing 80 compounds.

In this study, two plates (plates A and E) comprising 160 compounds were selected for initial screening based on assay throughput considerations, providing a representative subset for preliminary hit identification. These plates contain compounds spanning multiple chemical classes and disease indications, providing a manageable yet chemically diverse representative subset. Compound handling and dilution were performed according to MMV guidelines.^2^ Briefly, 1 mM parent stock solutions were prepared in 100% DMSO from the 10 mM master stocks supplied by MMV, aliquoted into 96-well plates, and stored at −20 °C. Working solutions were freshly prepared by diluting the intermediate stocks into autoclaved distilled water and subsequently into the assay medium to achieve a final compound concentration of 10 µM (1% DMSO). The two compounds selected for downstream mode-of-action studies, MMV667494 and MMV028694, were selected based on their sub-micromolar activity in secondary screening assays (Supplementary Material 1). Fresh supplies of these compounds were subsequently obtained from MMV, and 10 mM stock solutions were prepared in DMSO for all confirmatory and mechanistic experiments.

### Ethical approval

Ethical approval and informed content were not required for this study, as all experiments were conducted using established cell lines and did not involve human participants or animal subjects.

### Trypanosome cell cultures

Wild-type bloodstream-form *Trypanosoma brucei brucei* GUTat 3.1 cells in logarithmic growth phase were cultured at 37 °C and 5% CO_2_ in HMI-9 medium [35], supplemented with 10% fetal bovine serum and 1% penicillin-streptomycin.

### *In vitro* antitrypanosomal screening

Antitrypanosomal activity was assessed using a resazurin-based viability assay adapted from a previously described method [36]. This assay measures metabolic activity as a proxy for parasite viability. Screening was conducted in two phases: (i) a single-concentration primary screen to identify active compounds, and (ii) a dose-response secondary screen to determine IC_50_ values of the compounds. For the primary screen, a single-concentration assay (1 µM) was used to screen all compounds. Compounds were incubated with *T. b. brucei* cells (2 × 10^4^ cells/well) for 24 h, followed by the addition of alamarBlue dye solution (500 µM of resazurin sodium salt in PBS pH 7.3) and further incubation for 24 h. Fluorescence was measured at an excitation wavelength of 530 nm and an emission wavelength of 500 nm using a Varioskan™ multimode plate reader (Thermo Fisher Scientific). Antitrypanosomal activity in this context was defined as a reduction in resazurin fluorescence relative to untreated controls, indicating reduced metabolic activity. The assay was performed in four biological replicates. Untreated cells served as negative controls, while cells treated with 1 µM diminazene aceturate, a standard antitrypanosomal drug, served as positive controls. For secondary screening, selected compounds were subjected to two-fold serial dilution (maximum concentration: 1 µM) to determine the IC_50_ values. The upper concentration limit was capped at 1 µM to prioritize the identification of high-potency compounds with sub-micromolar activity. The assay was carried out as described for the primary screening. IC_50_ values were determined using a non-linear regression (four-parameter logistic model) in GraphPad Prism version 9. The experiments were conducted in three biological replicates, each with three technical replicates.

### *In vitro* mammalian cytotoxicity assay

Mammalian cytotoxicity was assessed using RAW 264.7 murine macrophages and HEK 293 human embryonic kidney cells in a resazurin-based cell viability assay. These cell lines were selected to provide a preliminary assessment of compound toxicity across immune and non-immune mammalian cell types. Cells were seeded at 1.5 × 10^4^ cells/well and incubated for 24 h in Dulbecco’s Modified Eagle’s Media (DMEM) before compound exposure. After 24-h treatment, alamarBlue dye solution (500 µM of resazurin sodium salt in PBS) was added, and fluorescence was measured after an additional 24 h. As with the antitrypanosomal assay, this assay reflects metabolic activity as a proxy for cell viability. The half-maximal cytotoxic concentration (CC_50_) values were determined using a non-linear regression (four-parameter logistic model) in GraphPad Prism version 9. The assay was conducted in three biological replicates, each with three technical replicates. Cells treated with phenylarsine oxide (PAO; Sigma-Aldrich) served as the positive control. Selectivity indices (SI = CC_50_/IC_50_) were calculated.

### Growth profiling

To assess effects on parasite proliferation, bloodstream-form *T. b. brucei* cells were treated with compounds at different concentrations (1 × IC_50_, 2 × IC_50_, and 4 × IC_50_). Cells were seeded at 2 × 10^5^ cells/mL, and viable parasites were counted every 24 h using a haemocytometer over a period of up to 4 days. Cells were subcultured daily to maintain densities at 2 × 10^5^ cells/mL. Cumulative cell numbers were calculated to account for daily dilution during subculturing, and doubling times were obtained directly from non-linear regression analysis (exponential growth model) in GraphPad Prism version 9, using the best-fit parameter output. Statistical comparison between the treated and untreated cells was made using an ordinary one-way ANOVA with Dunnett’s multiple comparison test. Diminazene aceturate-treated cells served as positive controls and untreated cells served as the negative control. Experiments were conducted in three biological replicates, each with three technical replicates.

### Annexin V/propidium iodide apoptosis assay

The mode of *T. b. brucei* cell death following treatment with MMV667494 and MMV028694 was investigated using the Annexin V-FITC apoptosis detection assay (Sigma-Aldrich). The assay was used to identify apoptosis-like phenotypes based on phosphatidylserine exposure and membrane integrity; however, it does not confirm classical apoptosis in *T. b. brucei.* The experiment was carried out following the manufacturer’s instructions. Briefly, cells (2 × 10^5^) were treated for 24 h at 1 × IC_50_, 2 × IC_50_, and 4 × IC_50_, except the untreated cells which served as the controls. Propidium iodide was added immediately before acquisition to discriminate membrane-compromised cells. Data were acquired using a BD FACS LSR Fortessa X-20 flow cytometer with BD FACSDiva software version 8. Data analysis was carried out using FlowJo software 10.7.1. Statistical analysis was performed using two-way ANOVA with Dunnett’s multiple comparison test in GraphPad Prism version 9. Three independent experiments (biological replicates) were conducted.

### Cell cycle analysis

Cell-cycle distribution was assessed using the Guava cell cycle reagent (EMD Millipore, Cat No 4500-0220). Cells were treated for 24 h at 1× IC_50_, 2 × IC_50_, and 4 × IC_50_, and fluorescence acquisition was performed using a BD FACS LSR Fortessa X-20 flow cytometer with BD FACSDiva software version 8. Data were analysed using FlowJo software 10.7.1, and statistical analysis was performed using two-way ANOVA with Dunnett’s multiple comparison test in GraphPad Prism version 9. Two independent experiments (biological replicates) were conducted.

### Mitochondrial membrane potential (TMRE) assay

Mitochondrial membrane potential (Δψm) was measured using tetramethylrhodamine ethyl ester (TMRE; Thermo Fisher Scientific, T669). This assay provided a population-level assessment of mitochondrial membrane potential. Cells (2 × 10^5^ cells/mL) were treated for 24 h at 1× IC_50._ Valinomycin (100 nM) and troglitazone (10 µM) were used as depolarization and hyperpolarization controls, respectively [37]. Fluorescence was acquired using a BD FACSCalibur (4-colour) cytometer with CellQuest Pro software. Data were analyzed using FlowJo software 10.7.1, and statistical analysis was performed using two-way ANOVA with Dunnett’s multiple comparison test in GraphPad Prism version 9. Two independent experiments (biological replicates) were carried out.

### MitoSOX red assay

Mitochondrial reactive oxygen species (ROS) production was quantified using the MitoSOX Red assay as a specific indicator of mitochondrial oxidative stress associated with compound-induced cytotoxicity. Bloodstream-form *T. b. brucei* cells were treated with MMV667494 and MMV028694 at 1 × IC_50_, 2 × IC_50_ and 4 × IC_50_ for 1h and 24 h to distinguish early versus delayed mitochondrial ROS responses. Following treatment, cells were incubated with MitoSOX Red according to the manufacturer’s protocol, and fluorescence intensity was measured by flow cytometry using a BD FACS LSR Fortessa X-20 flow cytometer equipped with BD FACSDiva software version 8.0.1. A minimum of 10,000 events per sample were acquired, and debris and doublets were excluded by forward- and side-scatter gating. Mean fluorescence intensity (MFI) values were calculated for each condition and normalized to untreated controls at the corresponding time point. Data analysis was performed using FlowJo software version 10.7.1, The assay was conducted in three biological replicates. Statistical analysis was carried out using repeated measures two-way ANOVA with Dunnett’s *post hoc* test. Fluoresence intensity was used as a measure of mitochondrial superoxide levels, indicative of mitochondrial oxidative stress.

### Intracellular ROS assay

To determine whether compound-induced cell death was associated with intracellular oxidative stress, intracellular ROS levels were measured using a fluorometric ROS detection kit (Sigma-Aldrich, Cat No MAK145). Live bloodstream-form *T. b. brucei* cells (2 × 10^4^ cells per well) were treated with MMV667494 or MMV028694 at 1 × IC_50_, 2 × IC_50_ and 4 × IC_50_ for 1h and 24 h, respectively. Following treatment, the cells were washed with PBS and incubated with 100 µL of the master reaction mix for 1 h at 37 °C and 5% CO_2_, protected from light. Fluorescence was measured using a Varioskan™ multimode plate reader at excitation 520 nm and emission 605 nm. Fluorescence values were background-subtracted and normalized to untreated controls at each time point prior to statistical analysis to account for time-dependent changes in cell density and metabolic activity. The assay was conducted in three biological replicates. Statistical analysis was performed using repeated-measures two-way ANOVA with Dunnett’s and Sidak’s *post hoc* test.

### Fixation of cells and fluorescent staining for microscopy

To assess mitochondrial morphology and kinetoplast–nuclear organization, cells were stained with MitoTracker™ Red CMXRos (Invitrogen, Thermo Fisher Scientific) before fixation. The Mitotracker stain served as a qualitative assessment of mitochondrial morphology. Following compound treatment at 1 × IC_50_, cells were incubated with MitoTracker dye according to the manufacturer’s recommendations and subsequently fixed in 4% formaldehyde, as previously described [31, 38]. Fixed cells were mounted onto poly-L-lysine-coated slides (Sigma-Aldrich) and allowed to adhere for 1 h, washed with PBS, and blocked with 100 mM of Tris-HCl in PBS (pH 7.5) for 10 min and washed again with PBS to reduce background fluorescence. Nuclear DNA was counterstained with Hoechst 33342 (Invitrogen, Cat No H3570) before the final PBS wash and mounting with Vectashield antifade mounting medium (Vector laboratories, Cat No H-1000).

### Confocal microscopy and image analysis

Fluorescence imaging was performed using a Zeiss Axio Observer Z1 microscope equipped with an LSM 800 confocal unit using a 63× oil-immersion objective (Plan-Apochromat 63x/1.4 Oil DIC M27). Image acquisition was carried out using Zen Blue 2.3 software, with fluorescence channels configured using the “smart setup” function for two-colour imaging. Hoechst 33342 and Mitotracker Red CMXRos were excited using the 405 nm (5 mW) and 561 nm (10 mW) laser lines, respectively, with emission collected at 410–546 nm and 590–700 nm. Detector saturation was avoided using the range-indicator function, and Z-stacks were acquired at 1 µm intervals with a pinhole size of 1 Airy unit. Images were acquired in CZI format and exported as TIFF files for analysis in ImageJ (Fiji). Linear adjustments to contrast and brightness were applied uniformly across conditions, and Z-stacks were visualized using maximum-intensity projections. Kinetoplast/nucleus (K/N) counts as well as cell and mitochondrial morphology profiling were carried out manually using cell counts. At least 500 cells per condition were analyzed across biological replicates. Statistical analysis was carried out using one-way ANOVA with Sidak’s *post hoc* test.

## Results

### Identification of two selective antitrypanosomal compounds for mode of action studies

**FIGURE 1:**
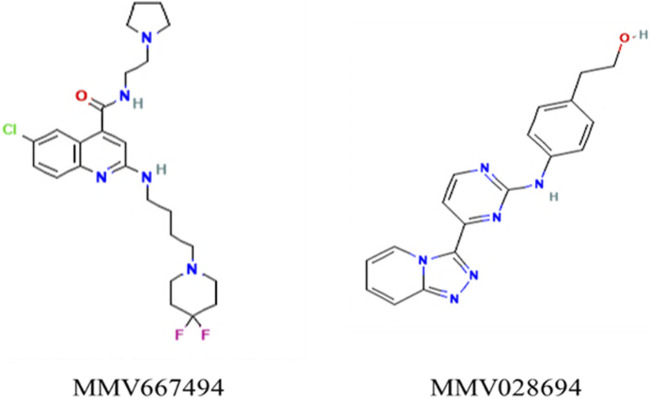
Chemical structures for the selected MMV compounds, MMV667494 and MMV028694. MMV667494 is 6-Chloro-2-[4-(4,4-difluoropiperidin-1-yl) butylamino]-N-(2-pyrrolidin-1-ylethyl) quinoline-4-carboxamide, while MMV028694 is 2-[4-[[4-([1,2,4] triazolo [4,3-a]pyridin-3-yl)pyrimidin-2-yl]amino]phenyl]ethanol. Both compounds are categorised as antimalarial compounds in the MMV pathogen box (https://www.mmv.org/mmv-open/pathogen-box/pathogen-box-supporting-information).

A total of 160 compounds from the MMV Pathogen Box (plates A and E compounds of theathogen box) were subjected to single-concentration *in vitro* antitrypanosomal phenotypic screening against bloodstream-form *T. b. brucei*. The primary screen was designed to be a one-point phenotypic assay to identify compounds that reduced parasite metabolic activity at 1 µM rather than to determine IC_50_ values. After this initial screen, 10 compounds demonstrating activity were identified for secondary screening. Secondary screening was performed using eleven two-fold serial dilutions (maximum concentration 1 µM), allowing the determination of IC_50_ values (Table 1). Two compounds, MMV667494 and MMV028694 (Figure 1), exhibited sub-micromolar potency (IC_50_ < 0.5 µM were prioritized for further analysis based on their potency. Both compounds are classified by MMV as antimalarial hits showed the most promising *in vitro* antitrypanosomal activity among the compounds tested (Table 1). To assess parasite selectivity, mammalian cytotoxicity was evaluated using RAW 264.7 murine macrophages and HEK 293 human kidney cells. Both MMV667494 and MMV028694 showed good selectivity toward *T. b. brucei*, with selectivity indices greater than 10 (calculated using 24 h CC_50_ and IC_50_ values), indicating their preferential toxicity toward trypanosomes relative to mammalian cells (Tables 1, 2; Supplementary Material 2).

**TABLE 1:** Antitrypanosomal activity and physiochemical properties of selected Pathogen Box compounds following secondary screening.

Compound ID	*T. b. brucei* IC_50_ ± SD (µM)	Disease set (as indicated by MMV)*	Total molecular weight*	Molecular formula*	cLogP*
MMV688889	0.67 ± 0.06	Tuberculosis	349.82	C_19_H_16_N_5_Cl	3.46
MMV688776	1.04 ± 0.07	Kinetoplastids	313.78	C_17_H_16_N_3_OCl	3.90
MMV676603	4.63 ± 0.66	Tuberculosis	431.39	C_17_H_16_N_3_O_5_F_3_S	1.29
MMV676401	3.85 ± 0.68	Tuberculosis	308.38	C_18_H_20_N_4_O	3.22
MMV688798	0.67 ± 0.08	Kinetoplastids	365.45	C_21_H_19_NO_3_S	3.48
**MMV667494**	**0.44 ± 0.05**	**Malaria**	**494.03**	**C**_**25**_ **H**_**34**_ **N**_**5**_ **OClF**_**2**_	**4.39**
**MMV028694**	**0.33 ± 0.03**	**Malaria**	**332.37**	**C**_**18**_ **H**_**16**_ **N**_**6**_ **O**	**2.12**
MMV689243	0.70 ± 0.08	Kinetoplastids	466.42	C_23_H_20_N_4_F_6_	4.26
MMV026313	0.50 ± 0.04	Malaria	237.26	C_14_H_11_N_3_O	1.92
MMV688415	2.85 ± 0.91	Kinetoplastids	433.54	C_26_H_31_N_3_O_3_	2.71

IC_50_ values (mean ± SD) were determined from three independent biological experiments, each performed in triplicate, using a resazurin-based viability assay in bloodstream-form *Trypanosoma brucei brucei*. Compounds were tested using two-fold serial dilutions with a maximum concentration of 1 µM. *Asterisked information (molecular weight, molecular formula, disease set classification, and cLogP values) was obtained from the MMV Pathogen Box Supplementary Material: https://www.mmv.org/mmv-open/pathogen-box/pathogen-box-supporting-information. IC_50_: half-maximal inhibitory concentration.

**TABLE 2:** Cytotoxicity and selectivity indices of MMV667494 and MMV028694 in mammalian cell lines.

Compound ID	*T. b. brucei* IC_50_ ± SD (µM)	RAW 264.7 CC_50_ ± SD (µM)	Selectivity index (CC_50_ RAW/IC_50_)	*HEK 293* CC_50_ ± SD (µM)	Selectivity index (CC_50_ HEK/IC_50_)
MMV667494	0.44 ± 0.05	5.50 ± 0.12	12.5	17.31 ± 2.5	39.3
MMV028694	0.33 ± 0.03	4.34 ± 0.38	13.2	8.12 ± 2.15	24.6
Diminazene aceturate	0.25 ± 0.00	7.33 ± 0.20	29.0	28.97 ± 7.37	115.9
Phenylarsine oxide	ND	0.57 ± 0.06	-	0.37 ± 0.03	-

CC_50_ values (mean ± SD) were determined in RAW, 264.7 murine macrophages and HEK, 293 human embryonic kidney cells using a resazurin-based viability assay after 24 h of compound exposure. Selectivity index (SI) was calculated as CC_50_ (mammalian cells) divided by IC_50_ (*T. b. brucei*). All experiments were performed in three independent biological replicates, each with three technical replicates. CC_50_, half-maximal cytotoxic concentration; SI, selectivity index.

### MMV667494 and MMV028694 display concentration- and time-dependent trypanocidal activity

**FIGURE 2:**
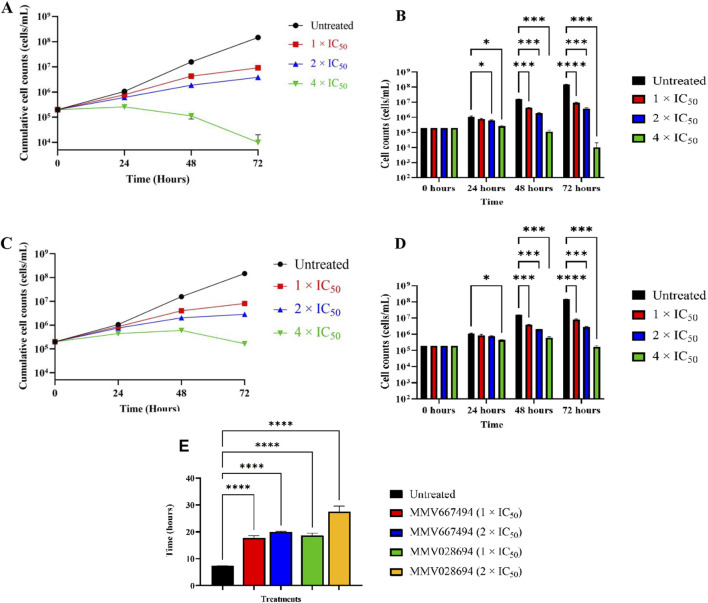
Growth inhibition and effects on doubling time of *T. b. brucei* following compound treatment. MMV667494 **(A,B)** and MMV028694 **(C,D)**. **(E)** Effect of compounds on doubling time. Cells were treated at 1 × IC_50,_ 2 × IC_50_ and 4 × IC_50_ for up to 72 h. Untreated cells served as controls. Data are averages of three independent experiments (biological replicates). Error bars show standard deviation. Statistical analysis was performed using ordinary one-way ANOVA with Dunnett’s multiple comparison test relative to untreated controls. Untreated cells served as controls. *p ≤ 0.05, ***p ≤ 0.001, ****p ≤ 0.0001. Cells were sub-cultured, as necessary, to maintain cell density below 2 × 10^6^ cells/mL.

The effect of both compounds on the proliferation of *T. b. brucei* cells was assessed by longitudinal growth profiling following treatment with increasing concentrations of MMV667494 (Figures 2A,B) and MMV028694 (Figures 2C,D). At 24 h, both compounds reduced cell numbers across all the tested concentrations (1 × IC_50_, 2 × IC_50_, and 4 × IC_50_), although statistical significance was reached only at higher concentrations - specifically MMV667494 at 2 × IC_50_ (p = 0.041), and 4 × IC_50_ (p = 0.014) and MMV028694 at 4 × IC_50_ (p = 0.026) (Figure 2). At 48 h and 72 h, both compounds produced dose-dependent reductions in parasite numbers, with statistically significant inhibition observed at all concentrations tested (p ≤ 0.0001) (Figures 2A,C). Notably, a net decline in parasite numbers was observed at 4 × IC_50_ after 72 h, indicating that both MMV667494 and MMV028694 killed a proportion of the trypanosome population under these conditions. At lower concentrations, parasite growth was inhibited without net loss of viable cells, consistent with a predominantly trypanostatic effect that transitions to trypanocidal activity at higher exposure, like previously reported for a series of adenosine analogues [39]. Consistent with these observations, cell doubling times were significantly prolonged following treatment. At 1 × IC_50_, doubling times increased to 17.7 h (MMV667494) and 18.7 h (MMV028694), while at 2 × IC_50_, doubling times further increased to 20.0 h and 27.6 h, respectively, compared with 7.4 h for untreated cells (Figure 2E). These findings confirm that both compounds markedly impair parasite proliferation in a concentration-dependent manner.

### MMV667494 and MMV028694 induce apoptotic-like phenotypes in a subset of treated cells

**FIGURE 3:**
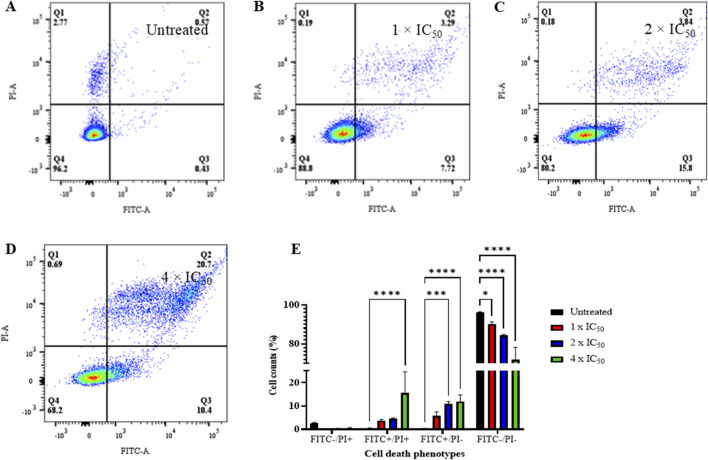
Annexin V/PI analysis of MMV667494-induced cell death in *T. b. brucei*. Representative pseudocolour two-parameter density plots showing Annexin V-FITC (x-axis) versus PI (y-axis) staining for **(A)** Untreated *T. b. brucei* cells, **(B)** MMV667494-treated (1 × IC_50_), **(C)** MMV667494-treated (2 × IC_50_), and **(D)** MMV667494-treated (4 × IC_50_) *T. b. brucei* cells. Trypanosomes (except the controls) were treated for 24 h. Quadrant interpretation: Viable cells are FITC-, PI- (lower left quadrant), early apoptotic-like cells are FITC+, PI- (lower right quadrant), and late apoptotic-like/necrotic cells are FITC+, PI+ (upper right quadrant). The FITC-, PI+ population (upper left quadrant) was classified as necrotic cells. **(E)** Shows the proportion of cells exhibiting different cell death phenotypes. Data represents SD from three independent experiments (biological replicates). Statistical analysis was performed using two-way ANOVA with Dunnett’s multiple comparison test relative to untreated and heat-treated controls. *p ≤ 0.05, ***p ≤ 0.001, ****p ≤ 0.0001. Note: The term apoptosis-like reflects phosphatidylserine externalization in trypanosomes, which lack canonical caspase-dependent apoptotic pathways.

**FIGURE 4:**
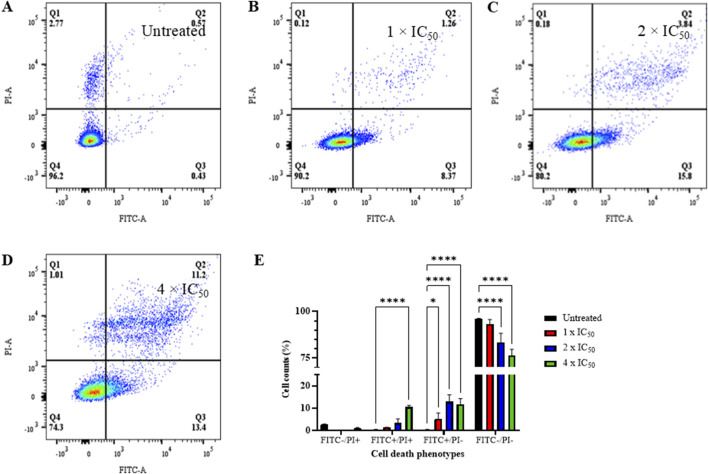
Annexin V/PI analysis of MMV028694-induced cell death in *T. b. brucei*. Representative pseudocolour two-parameter density plots showing Annexin V-FITC (x-axis) versus PI (y-axis) staining for **(A)** Untreated T. b. brucei cells, **(B)** 1 × IC_50_, **(C)** 2 × IC_50_, and **(D)** 4 × IC_50_. Trypanosomes (except the controls) were treated for 24 h. Quadrant interpretation: Viable cells are FITC-, PI- (lower left quadrant), early apoptotic-like cells are FITC+, PI- (lower right quadrant), and late apoptotic-like/necrotic cells are FITC+, PI+ (upper right quadrant). The FITC-, PI+ population (upper left quadrant) was classified as necrotic cells. **(E)** Shows the proportion of cells exhibiting different cell death phenotypes. Data represents SD from three independent experiments (biological replicates). Statistical analysis was performed using two-way ANOVA with Dunnett’s multiple comparison test relative to untreated and heat-treated controls. *p ≤ 0.05 and ****p ≤ 0.0001. Note: The term apoptosis-like reflects phosphatidylserine externalization in trypanosomes, which lack canonical caspase-dependent apoptotic pathways.

The annexin V-FITC/PI assay was used to investigate whether the observed trypanocidal cell death was associated with apoptosis-like or necrotic phenotypes. Phosphatidylserine is usually found in the inner leaflet of the plasma membrane of healthy cells. Upon initiation of apoptosis, phosphatidylserine is translocated to the extracellular membrane leaflet, making it easily accessible for annexin V-FITC to bind to it. Propidium iodide (PI), a nucleic acid binding dye, is a fluorescent indicator that is excluded from intact cells but enters when the plasma membrane becomes compromised. Annexin V-FITC/propidium iodide (PI) staining therefore allows to distinguish between viable (FITC-/PI-), early apoptotic-like (FITC+/PI-), late apoptotic-like (FITC+/PI+), and necrotic (FITC-/PI+) populations. The term “apoptotic-like” is used because *T. b. brucei* lacks canonical caspases, which are the classical apoptotic cell death signalling machinery, despite displaying hallmark features such as phosphatidylserine externalisation [40]. Treatment with MMV667494 for 24 h resulted in a significant increase in early apoptotic-like cells defined as increased Annexin V fluorescence but unchanged cell permeability measured with PI, at 2 × IC_50_ (10.9%, p = 0.0004) and 4 × IC_50_ (12.0%, p < 0.0001) compared with untreated controls 0.49%) (Figure 3). Late apoptotic-like cells were also significantly increased at 4 × IC_50_ (15.6%, p < 0.0001) with increased fluorescence on both channels, relative to untreated cells (0.52%) (Figures 3D,E). A similar pattern was observed for MMV028694, although significant increases in early apoptotic-like cells were already evident at 1 × IC_50_ (5.3%. p = 0.0193) (Figure 4). Despite these increases, the overall proportion of Annexin V-positive cells remained relatively modest indicating that apoptosis-like features were restricted to a subset of the population at the time points examined.

### Mitochondrial membrane depolarization occurs in a subpopulation of treated cells

**FIGURE 5:**
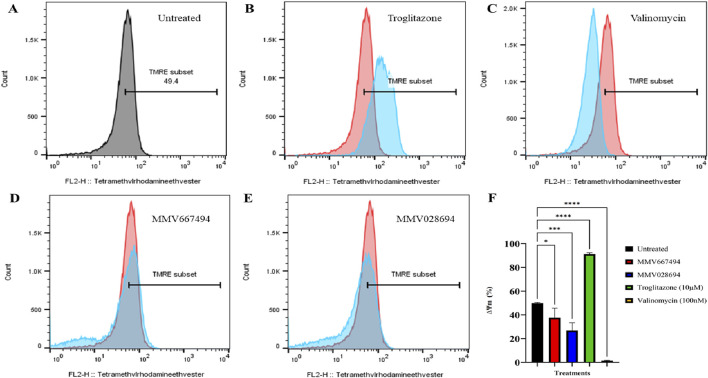
Effects of MMV667494 and MMV028694 on mitochondrial membrane potential in *T. b. brucei*. Representative histogram plots for trypanosomes stained with tetramethylrhodamine ethyl ester (TMRE). Histograms in red show untreated cells while the histograms in blue show respective treatment: **(A)** untreated, **(B)** troglitazone-treated (10 µM), **(C)** valinomycin-treated (100 nM), **(D)** MMV667494 (1 × IC_50_), and **(E)** MMV028694 (1 × IC_50_). Cells were treated for 24 h before assay **(F)** Shows the quantification of mean TMRE fluorescence intensity. Data represents mean ± SD from three independent biological replicates. Statistical analysis was performed using two-way ANOVA with Dunnett’s multiple comparison test relative to untreated and heat-treated controls. Valinomycin-treated cells served as the negative control (mitochondrial membrane depolarization) while troglitazone-treated cells served as the positive control (mitochondrial membrane hyperpolarization). Statistical analysis was performed using two-way ANOVA with Dunnett’s multiple comparison test, *p ≤ 0.05, ***p ≤ 0.001, ****p ≤ 0.0001. Note: depolarization was observed primarily in a subpopulation of cells, indicating heterogeneous mitochondrial responses rather than a uniform effect across population.

The mitochondrial membrane potential (ΔΨm) of *T. b. brucei* cells treated with MMV667494 and MMV028694 was determined by flow cytometry in TMRE-stained live cells exposed to a concentration of 1 × IC_50_ of the compounds for 24 h (Figures 5A–F). Both MMV667494 and MMV028694 treatments resulted in a statistically significant reduction in mean ΔΨm (p = 0.029 and p = 0.001 respectively). However, analysis of fluorescence distributions revealed that depolarization was confined to a distinct subpopulation of cells, while the majority retained mitochondrial membrane potential. This is evident from the observed low-fluorescence peak in treated samples (Figures 5D,E), suggesting that mitochondrial depolarization is associated with cells undergoing death or severe stress rather than a uniform effect across the population. Consistent with this interpretation, confocal microscopy of MitoTracker-stained cells showed largely preserved mitochondrial morphology in most treated cells, particularly following MMV667494 exposure (Figure 5F; Supplementary Figure S1). Control treatments behaved as expected: troglitazone induced mitochondrial hyperpolarization, while valinomycin caused complete depolarization (both p < 0.0001) (Figures 5A–C), in agreement with previous reports [37, 41].

### MMV667494 and MMV028694 increased mitochondrial ROS levels in a time-dependent manner

**FIGURE 6:**
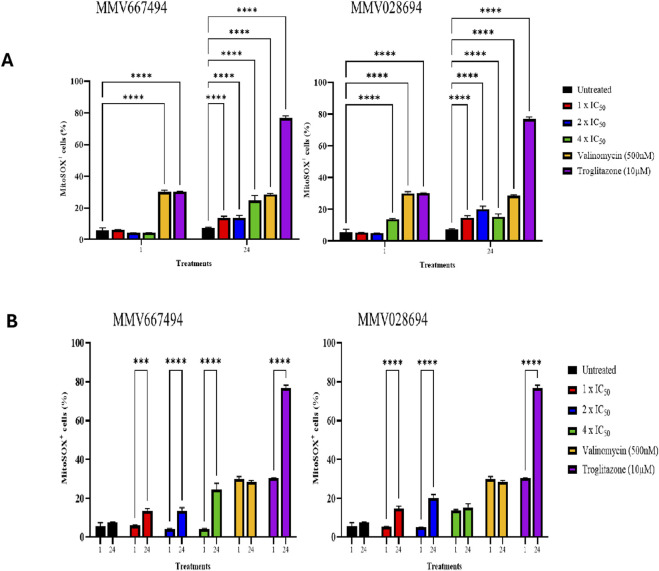
Effect of MMV667494 and MMV028694 on mitochondrial reactive oxygen species (mROS) levels in *T. b. brucei*. Mitochondrial superoxide levels were assessed using MitoSOX Red following 1-h and 24-h treatments. **(A)** mROS levels after 1 h of exposure and **(B)** mROS levels after 24 h of exposure. Cells were treated at 1 × IC_50_, 2 × IC_50_ and 4 × IC_50_. Data represents the mean mitochondrial superoxide levels for MMV667494-treated and MMV028694-treated trypanosomes stained with MitoSOX dye for three biological replicates. Statistical analysis was performed using repeated-measures two-way ANOVA with Dunnett’s multiple comparison test. Error bars indicate standard deviation. Untreated, valinomycin-treated and troglitazone-treated cells served as controls. ***p ≤ 0.001 and ****p ≤ 0.0001. Note: Increases in mROS were modest and time-dependent, suggesting association with downstream cellular stress rather than a primary mechanism of action.

The effect of MMV667494 and MMV028694 on mitochondrial levels of reactive oxygen species was determined using the MitoSOX red assay at 1 h and 24 h of treatment (Figure 6). MMV667494 did not significantly alter mitochondrial ROS levels at 1 h, whereas MMV028694 caused a significant increase only at 4 × IC_50_ (p < 0.0001) but not at lower concentrations (Figure 6A). After 24 h, both compounds significantly increased mitochondrial ROS levels at all concentrations tested, relative to untreated controls (Figure 6A). Time-course analysis revealed a significant increase (p < 0.0001) in mitochondrial ROS between 1 h and 24 h for MMV667494 at all concentrations (Figures 6A,B). For MMV028694, significant increases between time points were observed at 1 × IC_50_ and 2 × IC_50_ (p < 0.0001 for both), but not at 4 × IC_50_ (p = 0.76), likely reflecting advanced cell damage or loss of viable mitochondria at higher exposure (Figure 6B). Overall, the magnitude of ROS increases was modest, and the delayed onset suggests that mitochondrial oxidative stress is more likely a downstream consequence of compound-induced cellular effects rather than the primary cause of cell death.

### Intracellular ROS levels remain largely unchanged following treatment with MMV667494 and MMV028694

The intracellular levels of ROS in MMV667494-treated, MMV028694-treated and untreated cells were determined using a fluorescence-based commercially available kit as earlier described. The results obtained showed that neither MMV667494 nor MMV028694 significantly altered intracellular ROS levels at 1 h or 24 h across all tested concentrations, except for a just-significantly modest decrease observed at 1 h for MMV028694 at 4 × IC_50_ (p = 0.029) (Supplementary Material 4). As this effect was not sustained at 24 h, it is unlikely to be biologically meaningful. A similar increase in intracellular ROS was observed over time in both treated and untreated cultures, consistent with increased cell density and prolonged culture, leading to media changes, rather than compound-specific effects. These findings suggest that intracellular oxidative stress is not the primary mechanism underlying MMV667494- or MMV028694-induced parasite death.

### MMV667494 and MMV028694 disrupt cell-cycle progression without inducing widespread morphological abnormalities

**FIGURE 7:**
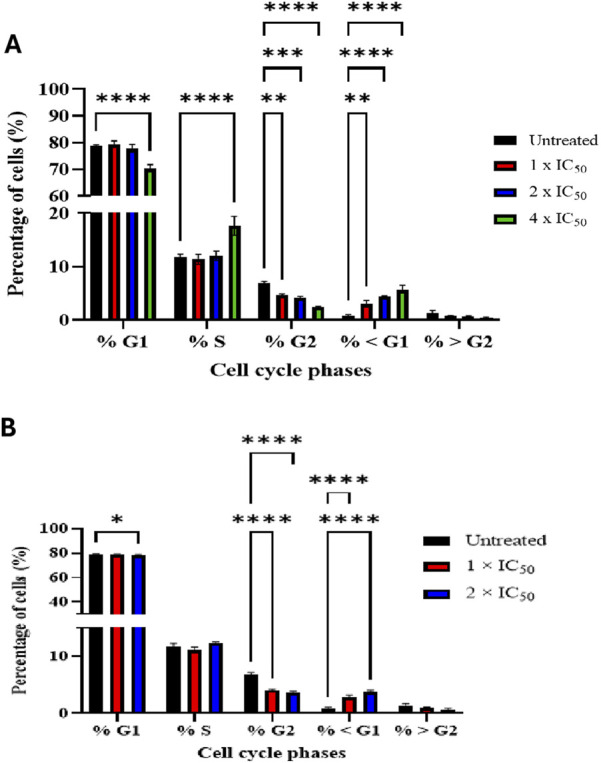
Effect of MMV667494 and MMV028694 on cell cycle progression in *T. b. brucei*. Cell cycle distribution was determined by flow cytometry following Hoechst staining for **(A)** MMV667494-treated cells and **(B)** MMV028694-treated cells. Untreated cells served as controls. Data represents mean ± SD from three biological replicates. Statistical analysis was performed using repeated-measures two-way ANOVA with Dunnett’s multiple comparison test relative to untreated controls. **p ≤ 0.01, ***p ≤ 0.001, ****p ≤ 0.0001. Note: Increased sub-G1 populations and altered G2/S distributions are consistent with disrupted cell cycle progression rather than a defined cell cycle arrest point.

Cell cycle effects were analyzed using DNA-content flow cytometry (Figure 7) and confocal microscopy (Supplementary Material 3, 5). Following MMV667494 treatment, a significant reduction in G1-phase cells was observed at 4 × IC_50_ (p < 0.0001), accompanied by a concomitant increase in S-phase cells (Figure 7A). G2-phase cells were significantly reduced at all concentrations tested, while cells with sub-G1 DNA content (<G1) increased in a dose-dependent manner, consistent with DNA fragmentation or aberrant division. Similarly, MMV028694 treatment resulted in the accumulation of <G1 cells and depletion of G2-phase cells i.e., with a completely replicated diploid set of chromosomes, at all concentrations (Figure 7B). A modest reduction in G1-phase cells was also observed, reaching statistical significance at 2 × IC_50_ (p = 0.0326). These profiles are consistent with disrupted cell cycle progression and accumulation of cells with abnormal DNA content. Overall, both compounds produced cell-cycle profiles consistent with slowed S-phase progression and impaired cell division, in agreement with the observed growth defects. The observations suggest that the compounds exerts a direct or indirect inhibitory action on DNA synthesis, likely not only prolonging S-phase but causing incomplete replicate of daughter chromosomes, leading to the highly significant drop in cells with fully replicated nuclei and associated increase in cells that contain less DNA than the normal diploid set of chromosomes (<G1 DNA content) after cell division, which would not be viable.

Confocal microscopy was used to validate the results obtained from the flow cytometry-based cell cycle progression assay. The confocal microscopy corroborated these findings at 1 × IC_50_ and 24 h of exposure. Both compounds increased the proportion of 0K1N cells (∼6% vs. 1.9% in controls) and 2K1N cells, while 1K2N and other aberrant K/N configurations remained rare (Supplementary Material 5A). MMV028694 uniquely increased the proportion of 1K0N cells, and rare events such as cojoined nuclei and cytokinesis failure (<1%) were observed (Supplementary Material 5B). Gross morphological distortions were limited to a very small fraction of treated cells and were not widespread (Supplementary Material 5C), indicating that cell death and growth inhibition primarily result from functional disruption of cell-cycle progression rather than widespread structural damage. Together, the most prominent changes in the N/K configuration therefore concerned the lack of an intact (visible) nucleus or kinetoplast at the point of cell division or thereafter. This is consistent with an impact of the treatments on DNA synthesis both of kinetoplast and nuclear DNA.

## Discussion

In this study, we aimed to identify antitrypanosomal hit compounds from a diverse synthetic library and to provide insights into their potential cellular effects using a cytology-based profiling approach. The compound set used - MMV’s Pathogen Box -comprises 400 drug-like molecules spanning multiple chemical classes, assembled based on known activity against pathogens responsible for neglected tropical diseases. Although the collection has supported repurposing efforts across diverse parasite systems, only a minority of Pathogen Box compounds were originally annotated with anti-kinetoplastid activity, leaving substantial space for systematic cross-pathogen exploration and repositioning.

Using a single-concentration phenotypic screening strategy followed by IC_50_ confirmation, we identified two compounds - MMV667494 and MMV028694 - with the most promising sub-micromolar activity against bloodstream-form *T. b. brucei* and selected them for further investigation. Both MMV667494 (a quinoline compound) and MMV028694 (a benzotriazole compound) were categorized by MMV as antimalarial hits, yet quinoline- and benzotriazole-related scaffolds have previously been associated with anti-trypanosomatid activity [42–46]. Prior studies have also reported antitrypanosomal activity for MMV028694 [42] and MMV667494 [29], though the IC_50_ values differ from those measured here - an expected outcome given that phenotypic potency estimates can vary across laboratories due to differences in parasite strains, media composition, incubation time, cell density, and growth rate [47, 48]. The present study extends these findings by providing a detailed phenotypic characterization of compound-induced cellular effects rather than assigning definitive molecular targets.

Published insights into possible targets for these scaffolds largely come from structural analogues characterized in malaria systems [29]. For MMV667494, quinoline-4-carboxamide analogues have been linked to inhibition of protein synthesis, particularly via interference with *Plasmodium* eEF2-dependent translation elongation [49–51]. Similarly, analogues related to MMV028694 have been associated with inhibition of c-Jun N-terminal kinase (JNK)-related signaling in *P. falciparum* [29]. While these data provide useful hypotheses, direct target equivalence cannot be assumed across parasites, particularly between apicomplexans and kinetoplastids. Therefore, the present study should be interpreted within the context of phenotypic evidence of compound-induced cellular perturbations occurring after treatment with MMV667494 and MMV028694, rather than definitive mechanistic targets.

Both MMV667494 and MMV028694 showed selective toxicity toward *T. b. brucei* relative to the mammalian cell lines tested, supporting their potential utility as starting points for lead optimization. Growth profiling revealed a pattern in which lower exposures primarily reduced proliferation rate. In contrast, higher exposure (4 × IC_50_ over 72 h) produced a net decline in parasite numbers, consistent with cidal activity under the conditions tested. Such transitions from trypanostatic-like growth suppression to trypanocidal outcomes at higher exposure are not uncommon [39] and may well reflect two separate mechanisms of action or, alternatively, inhibition of the same mechanism to a greater and more lethal extent. The markedly increased doubling time during the growth-inhibited phase indicates that even when parasites were not rapidly eliminated, cell-cycle progression and/or completion of cytokinesis was impaired.

The observed parasite death phenotype was apoptosis-like, supported by the increased proportion of Annexin V–positive cells (early and late apoptotic-like populations). In kinetoplastids, the term “apoptosis-like” is widely used because these organisms can display apoptosis-associated markers, including phosphatidylserine exposure, despite lacking canonical caspase machinery [40]. A notable feature of our dataset is the alignment between apoptotic-like populations and mitochondrial readouts. At 24 h and 1 × IC_50_, only a minority of cells showed marked mitochondrial membrane depolarization (TMRE-low), and this minority corresponded conceptually to the subset showing apoptotic-like features. Therefore, given the absence of canonical apoptotic machinery in trypanosomes, these findings should be interpreted cautiously. The pattern observed argues against an interpretation in which ΔΨm collapse is an early, uniform primary event. Instead, mitochondrial depolarization appears enriched in dying cells, consistent with a downstream hallmark of apoptosis-like death rather than a direct universal target effect.

Similarly, both compounds increased mitochondrial ROS (mROS) most clearly at 24 h, whereas global intracellular ROS did not increase in a compound-dependent manner. This distinction suggests that mitochondrial oxidative stress accompanies compound exposure and loss of viability but does not manifest as broad cytosolic oxidative stress and may not be the sole driver of parasite death. The timing also supports a model in which mROS elevation is either downstream of progressive cellular dysfunction, or part of an amplifying death cascade rather than the initiating process.

The most consistent and informative phenotype across assays was cell-cycle disruption, observed in DNA-content profiles and supported by microscopy-based K/N scoring. Both compounds increased sub-G1-like populations and reduced progression into normal G2/M distributions, consistent with impaired completion of a productive division cycle. Errors in the faithful replication of either nuclear or mitochondrial DNA, including the formation of dyskinetoplastic cells or cells with multiple nuclei, are a known trigger for apoptosis [52, 53]. The dataset shows that only a minority of cells displayed irregular kinetoplast/nucleus ratios or other cell cycle aberration–all consistent with the slow effect of the drug on *in vitro* cell growth. However, in choosing the moderate concentration and early exposure time, we believe we have captured the initial cellular events rather than only the late, indirect effects derived from the primary action of the drug on its target.

Overall, this study identified two promising antitrypanosomal compounds, MMV667494 and MMV028694, and characterized their effects on parasite growth and cellular physiology. Rather than defining a single molecular target, our findings indicate that both compounds induce a combination of growth and cell cycle inhibition, and apoptosis-like phenotypes, with mitochondrial perturbations occurring in a subset of affected cells. While MMV667494 and MMV028694 demonstrate promising *in vitro* activity and selectivity, several factors must be considered in evaluating their potential as drug discovery starting points. Both compounds originate from the Pathogen Box, a library curated for favourable drug-like properties, including physicochemical features compatible with oral bioavailability; however, further optimization and detailed pharmacokinetic and ADME profiling will be required to assess their suitability as lead compounds. In addition, the potential for resistance development remains an important consideration, and future studies should evaluate resistance selection and cross-resistance profiles. Importantly, the present study was conducted using *T. b. brucei* as a model organism, and validation against human-infective subspecies (*T. b. gambiense* and *T. b. rhodesiense*) will be necessary. Finally, *in vivo* efficacy, pharmacokinetics, and toxicity studies will be required to determine whether the observed *in vitro* activity translates into therapeutic potential.

## Data Availability

The original contributions presented in the study are included in the article/Supplementary Material, further inquiries can be directed to the corresponding author.

## Footnotes

1
https://www.mmv.org

2
https://www.mmv.org/mmv-open/pathogen-box/pathogen-box-supporting-information
