# Supplementary figures and images for: Phenotypic profiling of Pathogen Box compounds MMV667494 and MMV028694 in bloodstream-form Trypanosoma brucei brucei
Source: Exp Biol Med (Maywood). 2026 Jun 12;251:10979. doi: 10.3389/ebm.2026.10979 (PMC13303395; doi:10.3389/ebm.2026.10979)

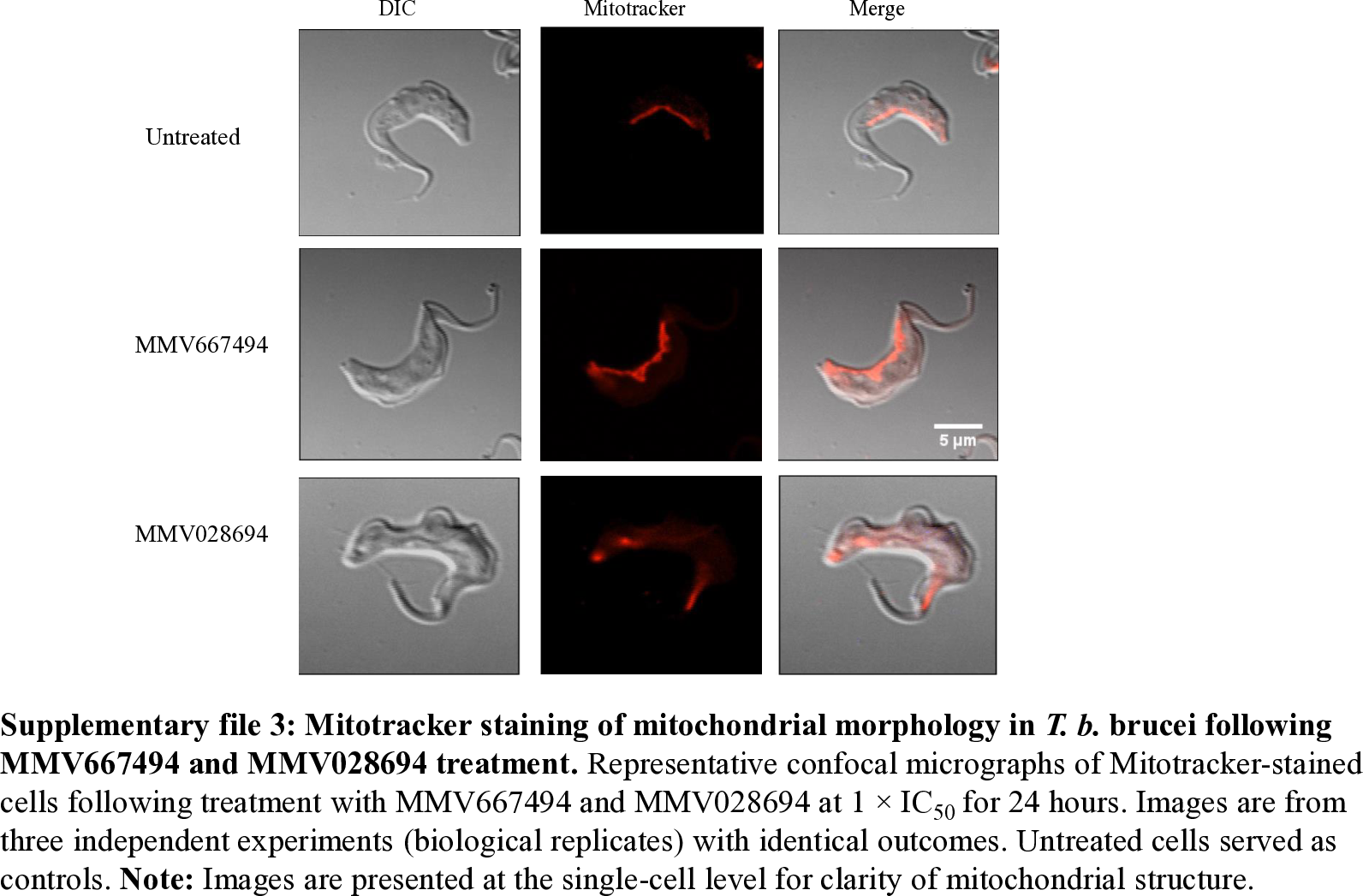

Supplement: Supplementary file 1 [file Image3.tif]

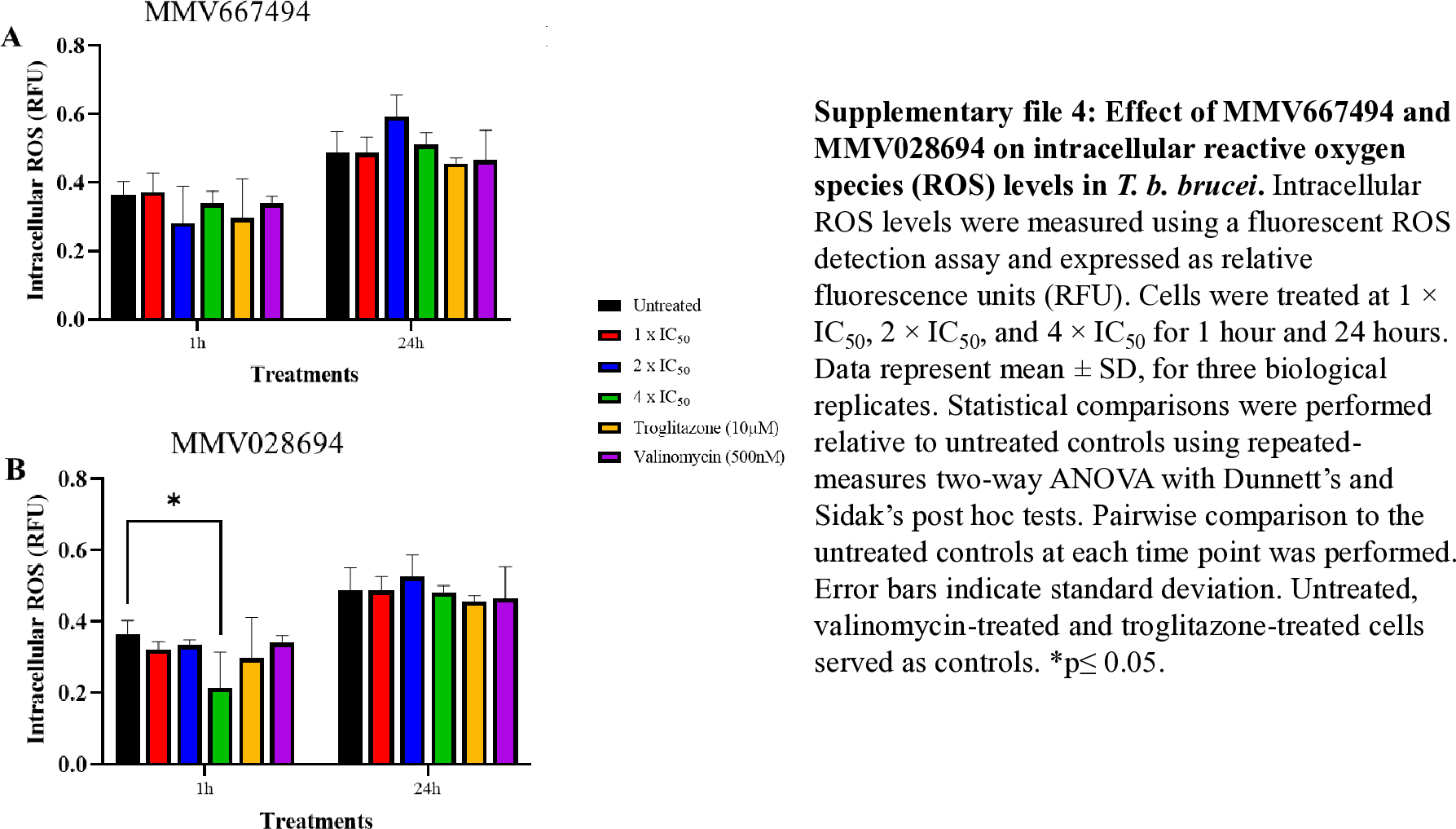

Supplement: Supplementary file 2 [file Image4.tif]

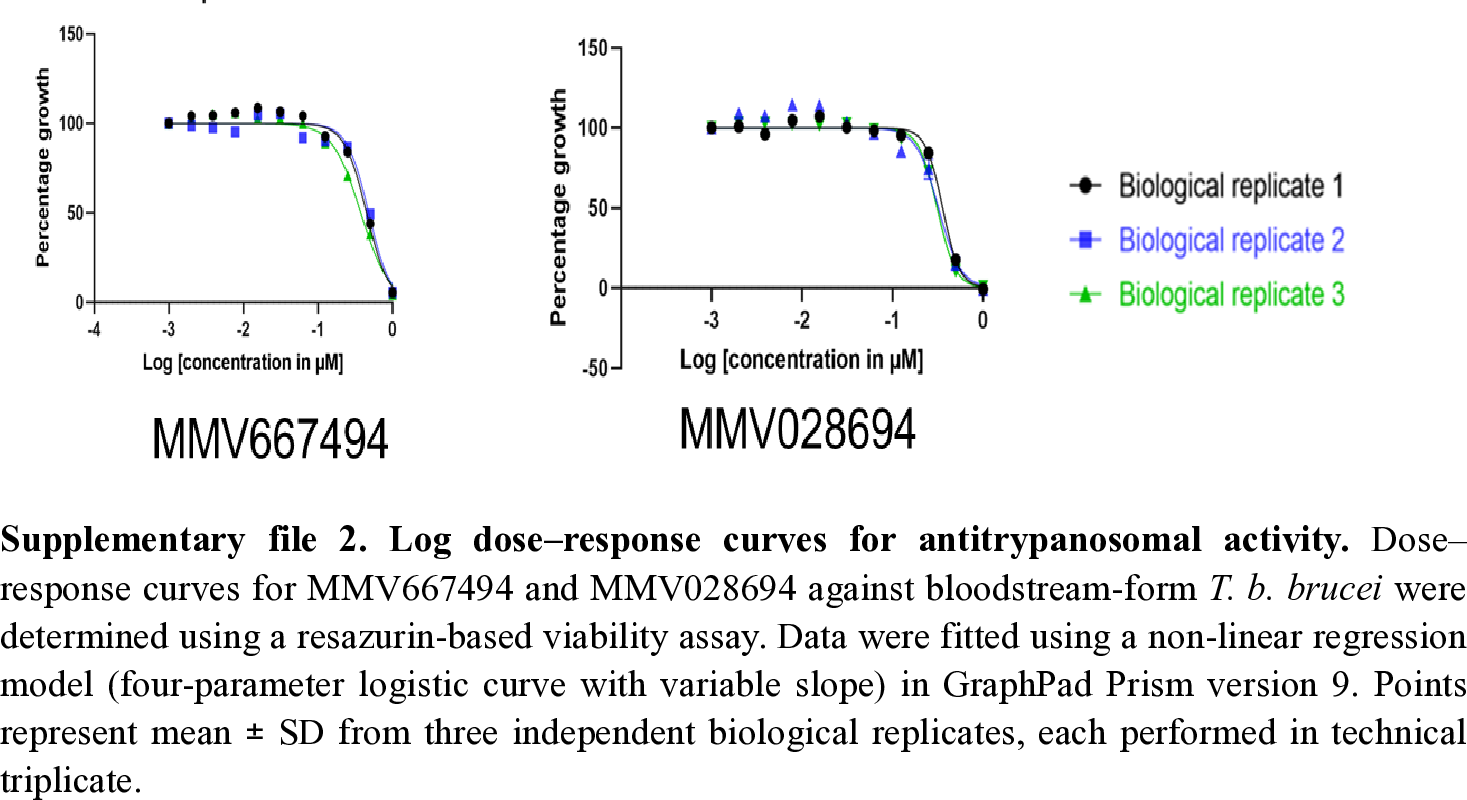

Supplement: Supplementary file 3 [file Image2.tif]

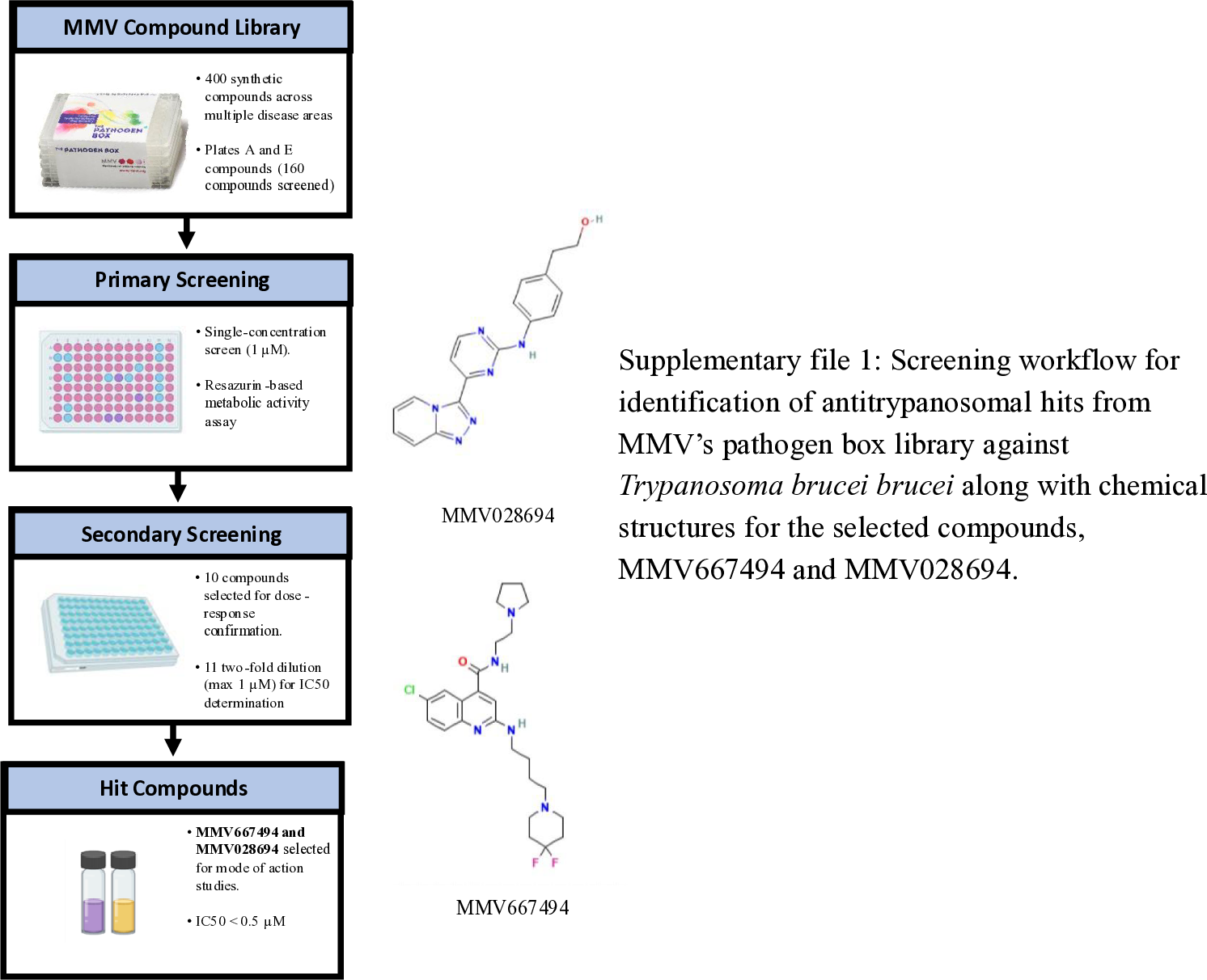

Supplement: Supplementary file 4 [file Image1.tif]

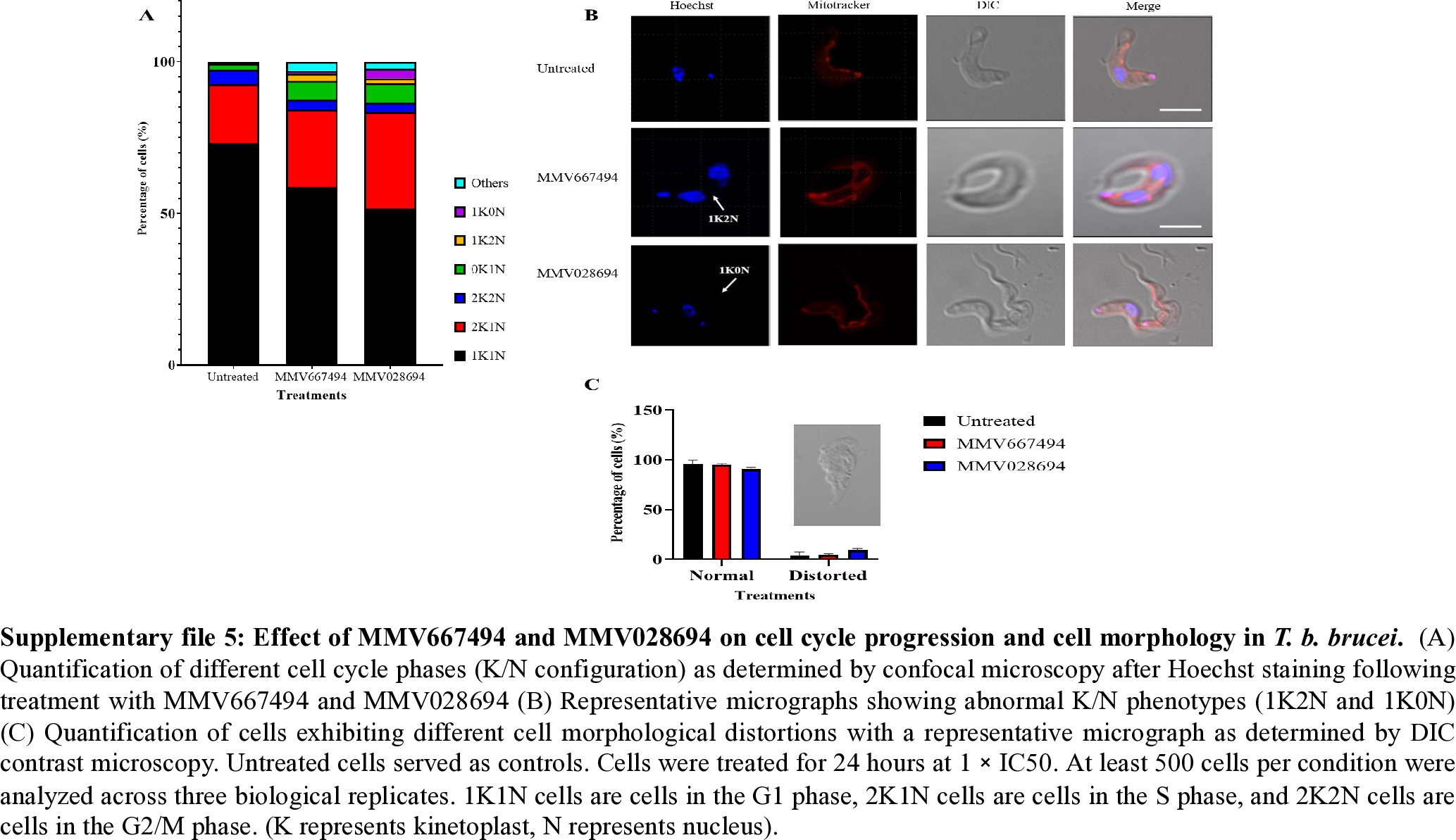

Supplement: Supplementary file 5 [file Image5.tif]
